# Effect of *Hirsutella sinensis* Fungus on the Hypothalamic-Pituitary-Adrenal Axis in Lewis Rats with Kidney-Yang Deficiency Syndrome

**DOI:** 10.1155/2020/5952612

**Published:** 2020-05-19

**Authors:** Lizong Zhang, Jiali Lang, Lu Jin, Lushuai Jin, Beibei Cao, Xia Shao, Mingsun Fang, Yueqin Cai, Xia Liu, Huiying Fu, Qiyang Shou

**Affiliations:** ^1^The Second Clinical Medical College, Zhejiang Chinese Medical University, Hangzhou 310053, China; ^2^Institute of Comparative Medicine, Zhejiang Chinese Medical University, Hangzhou, China; ^3^Department of Breast Surgery, Huzhou Central Hospital, Huzhou 313003, China

## Abstract

Kidney-yang deficiency syndrome (KYDS) is a classic syndrome in traditional Chinese medicine, which is mainly caused by damage to the hypothalamic-pituitary-adrenal (HPA) axis. *Hirsutella* sinensis fungus (HSF), an artificial substitute of *Cordyceps sinensis,* has been widely used in TCM. However, the effects and the possible mechanism of HSF on the HPA axis and corresponding KYDS have not yet been investigated. In this study, Lewis rats were used as a spontaneous KYDS model. HSF was intragastrically administered to the Lewis rats at two doses: low dose (1 g/kg) and high dose (2 g/kg). Body weight, temperature, and behavioral tests including grip strength, open field, and Morris water maze (MWM) tests were used to evaluate the KYDS symptoms. Enzyme-linked immunosorbent assay was used to detect the level of circulating adrenocortisol (ACTH), corticosterone (CORT), corticotropin releasing hormone (CRH), cyclic adenosine monophosphate (cAMP), and cyclic guanosine monophosphate (cGMP). In addition, mRNA expression of tumor necrosis factor alpha (TNF-*α*), interferon gamma (IFN-*γ*), interleukin 10 (IL-10), CRH, glucocorticoid receptor (GR), and mineralocorticoid receptor (MR) was detected by quantitative real-time polymerase chain reaction (Q-PCR). The Lewis rats were indicated to have KYDS symptoms and HSF treatment ameliorated these symptoms via enhancement of the HPA axis function, which was evidenced by the increased levels of CRH, ACTH, and CORT in serum and 17-OHCS in urine. HSF also significantly improved the expression of TNF-*α*, IFN-*γ*, and IL-2, secreted by Th1 cells, which might accelerate the activation of the immune system related to the HPA axis function. Thus, we conclude that HSF can alleviate KYDS symptoms in Lewis rats by regulating the HPA axis through accelerated immune system activation.

## 1. Introduction

Traditional Chinese medicine (TCM) employs a yin-yang balance as a conceptual framework of the human body; this is a crucial factor in controlling normal functions or homeostasis. Kidney-yang deficiency syndrome (KYDS) is a classic syndrome in TCM. Patients with KYDS show a profile of symptoms including soreness and weakness of the waist and knees, fatigue, hearing impairments, feeling cold throughout the body, and teeth looseness [1, 2]. Modern research has indicated that KYDS is characterized by disorders of multiple metabolic pathways. Damage to the hypothalamic-pituitary-adrenal (HPA) axis that includes the adrenal gland, thyroid, and gonads is the major pathological mechanism of KYDS [2, 3].

Since the first animal model of KYDS was established in the early 1960s, various animal models with a range of clinical manifestations similar to the characteristics observed in humans with KYDS have been developed [1]. Pertinently, Lewis rats that displayed a decreased capacity of glucocorticoid receptors (GRs) in the pituitary gland, lower concentrations of corticotropin releasing hormone (CRH) messenger ribonucleic acid (mRNA) in the hypothalamus, fewer adrenocortical cells in a similar adrenal weight, and lower adrenocorticotropic hormone (ACTH) and corticosterone plasma levels than Wistar rats responded to a variety of stimuli [4]. In addition, Lewis rats are susceptible to experimental encephalomyelitis, rheumatoid arthritis, and other autoimmune diseases closely related to KYDS. Therefore, the physiological characteristics of Lewis rats are similar to the clinical manifestations of KYDS; in the present study, Lewis rats are used as a spontaneous KYDS animal model.

*Cordyceps sinensis* has long been used as a valuable medicinal fungus with an overwhelming list of pharmacological effects in TCM [5, 6]. Owing to the rarity of *Cordyceps sinensis, Hirsutella sinensis* fungus (HSF), an artificial substitute produced by injecting *Cordyceps sinensis* into bat moth larvae and getting the sporophore in the fermentation process, has been used as an attractive substitute with similar pharmacological properties including anticancer, anti-inflammatory, and antioxidant effects [7–9]. HSF significantly enhances phagocytosis via uninucleate macrophages, humoral immunity, and cellular immunity of mice in a clear dose-dependent manner [10]. Moreover, HSF could regulate a tumor necrosis factor alpha (TNF-*α*), interleukin 1 beta (IL-1*β*), transforming growth factor beta 1 (TGF-*β*1), and interferon gamma (IFN-*γ*) imbalance [11]. However, the effects and the possible mechanism of HSF on the HPA axis and corresponding KYDS have not yet been reported.

The hypothalamus is the integration center of the nerve-endocrine-immune network. The immune system possesses neuroendocrine hormone receptors and synthesizes neurotransmitters and endocrine hormones. Cytokines produced by the immune system can affect the central nervous system; the central nervous system can synthesize cytokines and their receptors and react to them [12, 13]. The present study aimed to investigate whether HSF could improve KYDS symptoms by regulating the function of the HPA axis in Lewis rats and to examine the possible mechanisms of this effect.

## 2. Materials and Methods

### 2.1. Chemicals and Reagents

HSF was obtained from Hangzhou KSBIO Science and Technology Co., Ltd. (Hangzhou, Zhejiang, China).

### 2.2. Experimental Animals and Treatment

Ten specific pathogen-free (SPF) male Wistar rats (7-8 weeks old, weighing 220–240 g) were purchased from the Shanghai SLAC Laboratory Animal CO. LTD (Shanghai, China) (Production Permit: SCXK (Hu) 2012-0002). Thirty SPF male Lewis rats (7-8 weeks old, weighing 240–260 g) were purchased from the Beijing Vital River Laboratory Animal Technology Co., Ltd. (Production Permit: SCXK (Jing) 2012-0001). The animal protocol experiment was approved by the Ethics Committee of Zhejiang Chinese Medical University. All applicable international, national, and/or institutional guidelines for the care and use of animals were followed. The animals were acclimatized for 1 week to a constant temperature (22°C) and humidity (72%) along with a 12 h light/dark cycle. The rats had free access to a standard laboratory diet and were provided water ad libitum; all experimental animals were given humane care according to the 3Rs principle [14].

Ten Wistar rats were used as a healthy control group (Wistar control group) and received 10 mL/kg saline. Thirty Lewis rats were randomly assigned to 3 groups (*n* = 10 per group): the Lewis control group (10 mL/kg saline, intragastric administration (ig)), Lewis + HSF low dose group (1 g/kg HSF, ig), and the Lewis + HSF high dose group (2 g/kg HSF, ig). Lewis rats were regarded as the spontaneous KYDS model because of their congenital deficiency of the HPA axis. The saline and drugs were administered on the first day of the experiment and last up to three weeks.

### 2.3. Euthanasia and Sample Collection

At the end of the experiments, the rats were sacrificed by euthanasia and the blood and tissue were collected. Euthanasia was carried out by an overdose of CO_2_ in a closed plastic chamber, followed by exsanguination. This method meets the recommendations of Ethics Committee of Zhejiang Chinese Medical University. Blood was withdrawn for serum analysis via an intracardiac catheter. Immediately following euthanasia, the animals were carefully dissected to collect the kidney.

### 2.4. General Observations

While testing the model rats, their general characteristics including body weight, anal temperature, food and water intake, urination, defecation, hogback presence, and irritability were recorded. At the end of the tests, the autonomous activities of rats were observed; all rats were placed in a quiet and dark environment for 1 minute; then, their autonomous activities were recorded for 5 minutes with a multifunctional event recorder (ZhengHua, AnHui, China).

### 2.5. Grip Strength Test

Fore-limb grip strength was measured as maximum tensile force using a rat Grip Strength Meter (Columbus Instruments, Columbus, OH) with a sensor range of 0–5,000 grams (g) and accuracy of 0.15%. The maximum of three pulls of the Grip Strength Meter conducted between 9 AM and 11 AM was recorded; the test was performed daily over three consecutive days and the resulting three daily maximum values were averaged [15].

### 2.6. Open-Field Test

The open-field apparatus, a square box (80 cm × 80 cm × 40 cm) without a cover with the floor painted black, was divided into 25 equal pieces by white lines. In a 5-minute test phase, an individual rat was placed in the central area of the bottom of the box. The number of pieces the rat walked through was recorded as its horizontal movement to show its activity. Vertical movements were recorded as the frequency of rearing to show the curiosity of the rats regarding the new environment. In addition, grooming, defecation, urination, and other behaviors were noted. The test environment was kept dark and quiet to reduce potential anxiety caused by the environment. The arena floor and the inner wall were cleaned thoroughly before and after each trial with a moist cloth, then with 90% ethanol to remove any residue of odorants and marks left by the animal.

### 2.7. Morris Water Maze Test

All rats were tested for the capacity of learning and memory in the Morris water maze (MWM) after the open-field test. The MWM apparatus consists of a dark gray circular pool (80 cm in diameter and 50 cm in height), a camera attached to a computer monitoring screen, a Smart-MASS system (Panlab, Spain) that recorded all the performances, and a metal platform (10 cm in diameter) immersed in water (22 ± 0.5°C) about 2 cm. All rat activity was tracked and could be displayed by the computer software. After the allowed time or if the animal escaped onto the platform, the computer stopped tracking and recorded the swimming trajectory; then, it automatically calculated the distance that animals swam in the pool and the time required to find the platform (latency).

In the MWM, the vision and motor abilities of the animals could be evaluated by visible platform-seeking behavior. The water surface was equally divided into 4 quadrants (I, II, III, and IV); the platform was fixed in the center of quadrant II. Before the formal test, rats were gently placed in the water and allowed to perform training and swim over the wall to get used to the tank environment and find the platform. During training, the rats were taken out of the water immediately after they climbed onto the platform; otherwise, if the rats could not find the platform in 2 minutes, they would be placed on the platform to rest for 20 s. The rats were trained twice daily for 4 consecutive days; then the classic MWM test was conducted. Each rat was placed in the pool from the same location and the latency was recorded. Next, the platform was removed and the rats were placed in the same quadrant to perform a space search test during which the distance to the original platform position, total path percentage, duration in the original platform position, and number of times the rat entered the original platform position (as an indicator of memory) were recorded. The test was conducted in a quiet room with sound insulation and the positions of various laboratory objects such as pools, fluorescent lamps, and squirrel cages remained unchanged.

### 2.8. Enzyme-Linked Immunosorbent Assay Detection

Rats in each group were anesthetized by an intraperitoneal injection of 3% sodium pentobarbital solution. Heart blood was collected and placed in centrifugal tube; the serum was subsequently separated using a refrigerated centrifuge at 4°C and 3000 rpm for 10 min. Urine samples were collected from metabolic cages and centrifuged at 4°C for 10 min at 5000 rpm for a 17-hydroxycorticosteroids (17-OHCS) test. Levels of ACTH, corticosterone (CORT), CRH, cyclic adenosine monophosphate (cAMP), and cyclic guanosine monophosphate (cGMP) in serum as well as 17-OHCS in urine were detected by enzyme-linked immunosorbent assay (ELISA) kits (Biovol Technologies, Shanghai, China).

### 2.9. RNA Extraction and Messenger RNA Expression Analysis by Quantitative Real-Time Polymerase Chain Reaction

The total RNA from the cortex renis of the rats was extracted using a TRIzol reagent (TaKaRa, Otsu, Japan). Complementary deoxyribonucleic acid (cDNA) was synthetized by reverse transcriptase with the PrimeScript RT reagent kit according to the manufacturer's protocol (TaKaRa). Quantitative polymerase chain reaction (qPCR) was performed using a fluorescence qPCR instrument (Bio-Rad, USA). The qPCR reaction was conducted with the following reagents: 12.5 *µ*L of 2 × SYBR, 0.5 *µ*L of each primer (10 *µ*M), 2 *µ*L of template cDNA, and enough RNase-free double distilled H_2_O to make the total volume 25 *μ*L. Cycling conditions were as follows: 94°C for 30 s, 40 cycles for 10 s at 95°C, and 30 s at 55°C. Fluorescence detection was achieved at 60°C; relative quantification was accomplished by the comparative CT (2^−△△CT^) method. The primer sequences are shown in Table 1.

### 2.10. Statistical Analysis

Differences between the mean values of normally distributed data were assessed using a one-way analysis of variance (ANOVA) using the statistical package for the social sciences software, version 19.0 (SPSS Inc., Chicago, IL, USA). For the comparison of 2 groups, Student's *t*-test was used and *P* < 0.05 was considered statistically significant. All data were expressed as the mean ± the standard deviation.

## 3. Results

### 3.1. Effect of *Hirsutella sinensis* Fungus on Body Weight and Temperature in Lewis Rats

**Figure 1:**
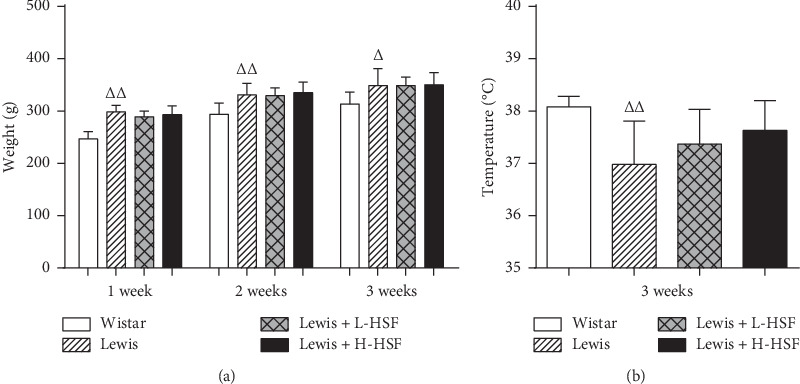
Changes in weight among groups and the temperature in the 3^rd^ week.

Weight loss symptoms are always present in KYDS animal models; hence, the effects of HSF on the weight and temperature of Lewis rats were evaluated. Due to the differences in animal strains, the weight of Lewis rats was significantly higher than that of Wistar rats at the same ages (*P* < 0.01). There was no significant change in the body weight of Lewis rats after HSF administration (*P* > 0.05). Compared with the Wistar control group, the rectal temperature in the Lewis group was significantly lower than that in Wistar rats (*P* < 0.01); this is consistent with the clinical symptoms of KYDS. The rectal temperature of HSF treatment groups had a similar dose-dependent increasing trend compared with the Lewis control group, though there was no significant difference (*P* > 0.05, Figure 1).

A comparison of medication administration groups and corresponding kidney-yang deficiency syndrome model groups is shown. The error bars represent the standard deviation of measurements for rats in each group (*n* = 10). ^Δ^*P* < 0.05; ^ΔΔ^*P* < 0.01; Wistar indicates Wistar control rat group; Lewis, Lewis control rat group; Lewis + L-HSF, Lewis rats administered a low dose of HSF (1 g/kg); and Lewis + H-HSF, Lewis rats administered a high dose of HSF (2 g/kg).

### 3.2. *Hirsutella sinensis* Fungus Ameliorated Kidney-Yang Deficiency Syndrome Behaviors in Lewis Rats

**Figure 2:**
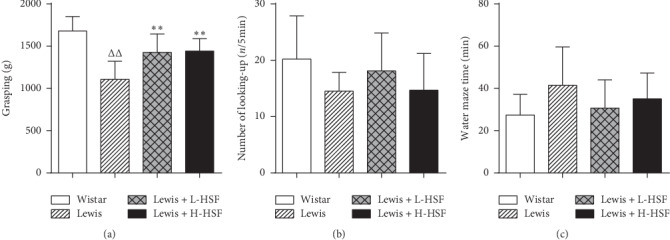
Changes of the grip strength, autonomic activity, and memory with grip strength, open field, and MWM tests.

The grip strength, open field, and MWM tests were used to evaluate the motor and cognitive function because KYDS animals typically show signs of exhaustion and fatigue such as decreased activity, slow reaction, and listlessness that reflect the symptoms KYDS patients. In the grip strength test, the grip strength of the Lewis control rats was significantly lower than that of Wistar control rats (*P* < 0.01); this is in agreement with the clinical malaise symptoms of KYDS. HSF administration significantly improved the grip strength of Lewis rats (*P* < 0.01, Figure 2(a)). In the open-field test, the Lewis control group looked up fewer times compared with Wistar control rats. After HSF administration, the looking-up behavior of Lewis rats improved, but there was no significant difference (*P* > 0.05, Figure 2(b)). Lewis rats took longer to reach the platform than Wistar control rats in the MWM test, indicating that the memory ability of Lewis rats was lower. HSF administration could shorten the time required for Lewis rats to reach the platform but did not lead to a significant difference (*P* > 0.05, Figure 2(c)). These results signify that Lewis rats indeed have KYDS symptoms and that they can be ameliorated by HSF administration.

In the comparison of model groups and Wistar normal control group, ^Δ^ indicates *P* < 0.05 and ^ΔΔ^ indicates *P* < 0.01. In the comparison of medication administration groups and corresponding model groups, ^*∗*^ denotes *P* < 0.05 and ^*∗∗*^ denotes *P* < 0.01. Wistar, Wistar control rat group; Lewis, Lewis control rat group; Lewis + L-HSF, Lewis rats administered a low dose of HSF (1 g/kg); and Lewis + H-HSF, Lewis rats administered a high dose of HSF (2 g/kg).

### 3.3. Effect of *Hirsutella sinensis* Fungus on Terminal Tissue Weights in Lewis Rats

**Figure 3:**
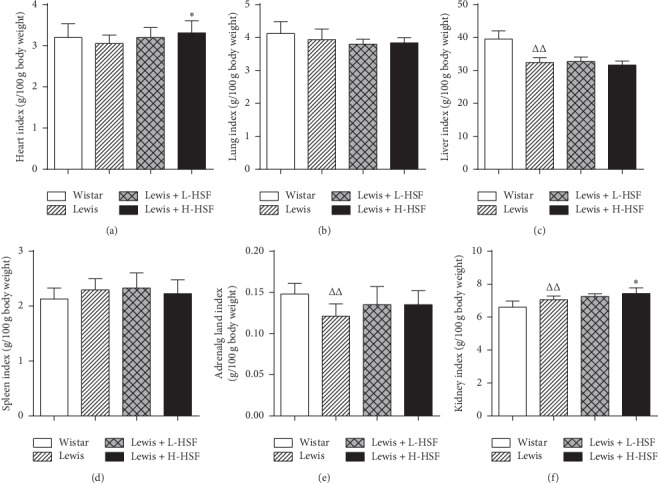
Changes of organ indexes in the KYDS model and control rats.

It is important to determine the main organ indexes in experimental animals as this information can reflect the degree of visceral lesions, to some extent, such as edema, hyperplasia, and atrophy. Compared with the Wistar control group, the liver and adrenal gland indexes of Lewis control rats were significantly lower and the kidney index was significantly increased (*P* < 0.01, Figures 3(c), 3(e), and 3(f)). After HSF administration, the heart and kidney indexes of Lewis rats increased significantly (*P* < 0.05, Figures 3(a) and 3(f)); the adrenal gland index also increased but without significance (*P* > 0.05, Figure 3(e)). The decrease in the adrenal gland index might be associated with an adrenocortical insufficiency.

In the comparison of the model groups and the Wistar control group, ^Δ^ indicates *P* < 0.05 and ^ΔΔ^ indicates *P* < 0.01. In the comparison of the medication administration groups and the corresponding model groups, ^*∗*^ indicates *P* < 0.05 and ^*∗∗*^ indicates *P* < 0.01. KYDS indicates kidney-yang deficiency syndrome; Wistar, Wistar control rat group; Lewis, Lewis control rat group; Lewis + L-HSF, Lewis rats administered a low dose of HSF (1 g/kg); and Lewis + H-HSF, Lewis rats administered a high dose of HSF (2 g/kg).

### 3.4. Effect of *Hirsutella sinensis* Fungus on the Hypothalamic-Pituitary-Adrenal Axis in Lewis Rats

**Figure 4:**
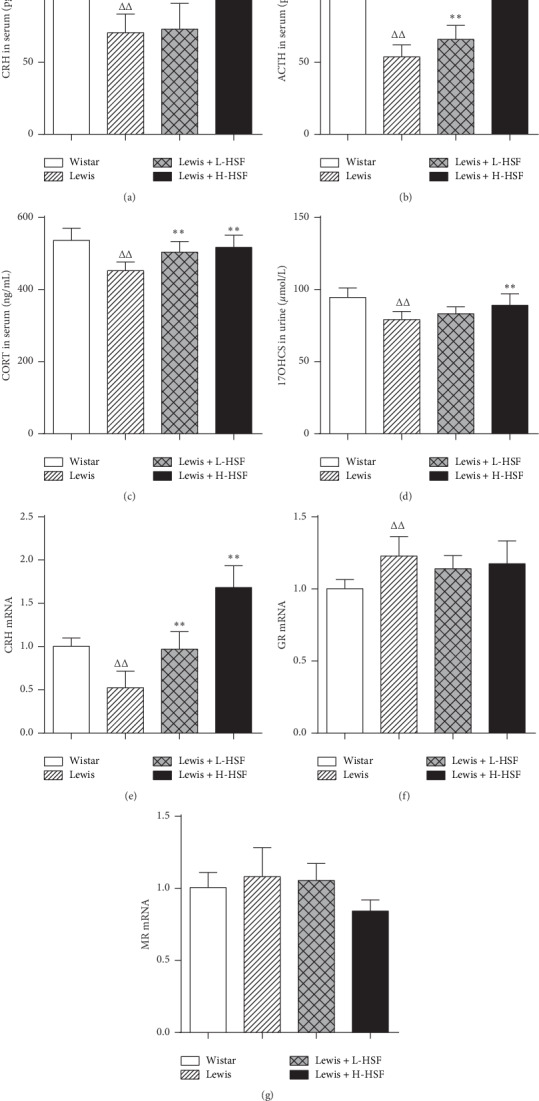
Changes of ACTH, CORT, and CRH in serum; 17-OHCS in urine; and CRH, GR, and MR mRNA in the hypothalamus.

A change in HPA axis function is widely regarded as the main pathological phenomenon of KYDS. Compared with the normal Wistar group, the levels of serum ACTH, CRH, CORT, and urine 17-OHCS in Lewis rats were significantly lower (*P* < 0.01), indicating that the CRH-ACTH-CORT axis (HPA axis) in Lewis rats spontaneously exhibited hypofunction without intervention. After HSF administration, serum ACTH and CORT and urine 17-OHCS levels increased significantly (*P* < 0.01); notably, there was a dose-dependent correlation. CRH levels tended to increase, but there was no significant difference (*P* > 0.05, Figure 4(a)–4(d)).

Compared with the Wistar control group, GR mRNA expression in the hypothalamus of Lewis rats increased significantly (*P* < 0.05), mineralocorticoid receptor (MR) mRNA expression in the hypothalamus increased but without significance (*P* > 0.05), and CRH mRNA expression in the hypothalamus decreased significantly (*P* < 0.01), indicating the hypofunction of the HPA axis in Lewis rats. After HSF administration, CRH mRNA in the hypothalamus was significantly increased (*P* < 0.01, Figure 4(e)); GR and MR mRNA decreased but without significance (*P* > 0.05, Figures 4(f) and 4(g)). Compared with Wistar rats, the HPA axis function in Lewis rats decreased significantly. HSF treatment enhanced the HPA axis function in Lewis rats, and the effect was dose dependent.

In the comparison of model groups and the Wistar control group, ^Δ^ indicates *P* < 0.05 and ^ΔΔ^ signifies *P* < 0.01. In the comparison of medication administration groups and corresponding model groups, ^*∗*^ indicates *P* < 0.05 and ^*∗∗*^ signifies *P* < 0.01. ACTH indicates adrenocortisol; CORT, corticosterone; CRH, corticotropin releasing hormone; 17-OHCS, 17-hydroxycorticosteroids; GR, glucocorticoid receptor; MR, mineralocorticoid receptor; mRNA, messenger ribonucleic acid; Wistar, Wistar control rat group; Lewis, Lewis control rat group; Lewis + L-HSF, Lewis rats administered a low dose of HSF (1 g/kg); and Lewis + H-HSF, Lewis rats administered a high dose of HSF (2 g/kg).

### 3.5. Effect of *Hirsutella sinensis* Fungus on Immunologic Factors in Lewis Rats

**Figure 5:**
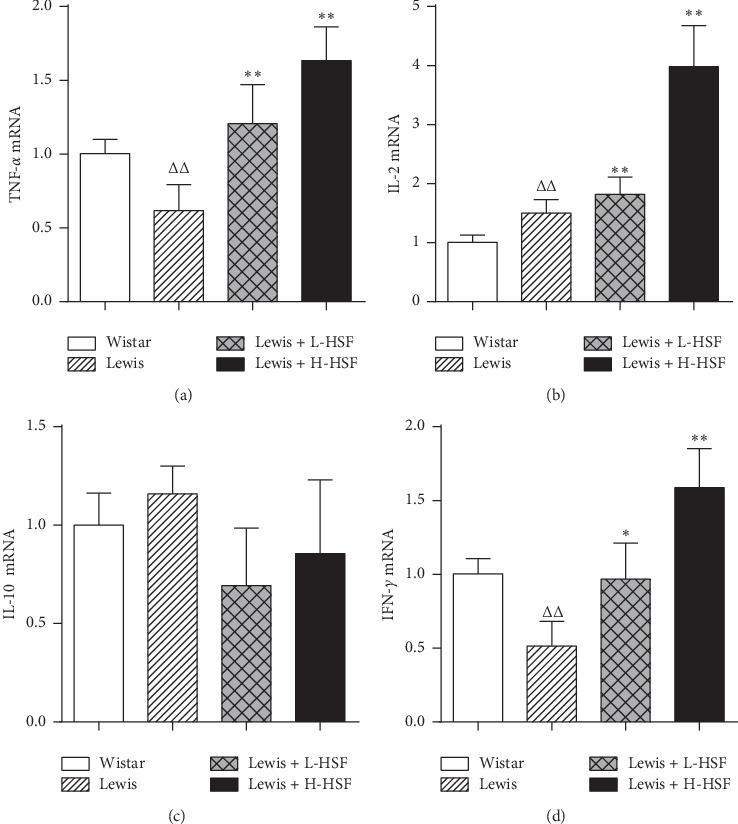
Changes of TNF-*α*, IL-2, IFN-*γ*, and IL-10 mRNA in the hypothalamus.

Immune hypofunction is the main manifestation of KYDS. Compared with Wistar control rats, the production of TNF-*α* and IFN-*γ* in the hypothalamus of Lewis rats decreased significantly (*P* < 0.01), while the production of interleukin 2 (IL-2) in the hypothalamus increased significantly (*P* < 0.01). After HSF administration, the levels of TNF-*α*, IFN-*γ*, and IL-2 in Lewis rats treated with HSF increased significantly (*P* < 0.05; *P* < 0.01), while the levels of IL-10 in the hypothalamus decreased without a significant difference (*P* > 0.05, Figures 5(a)–5(d)). Thus, HSF treatment can increase the level of cytokines secreted by immune cells, thereby enhancing the immune function of Lewis rats.

In the comparison of model groups and the Wistar control group, ^Δ^ indicates *P* < 0.05 and ^ΔΔ^ signifies *P* < 0.01. In the comparison of medication administration groups and corresponding model groups, ^*∗*^ indicates *P* < 0.05 and ^*∗∗*^ signifies *P* < 0.01. TNF-*α* indicates tumor necrosis factor alpha; IFN-*γ*, interferon gamma; IL-2, interleukin 2; IL-10, interleukin 10; mRNA, messenger ribonucleic acid; Wistar, Wistar control rat group; Lewis, Lewis control rat group; Lewis + L-HSF, Lewis rats administered a low dose of HSF (1 g/kg); and Lewis + H-HSF, Lewis rats administered a high dose of HSF (2 g/kg).

## 4. Discussion

There are 2 main types of KYDS animal models: etiological and pathological. Pathological models are widely used; they mainly use drugs or surgical methods to make experimental animals (usually rats) exhibit manifestations similar to KYDS in weeks. The pathological basis of KYDS is HPA axis dysfunction; drugs or surgical operations can affect the function of the axis. Compared with the pathological model, the etiological model is closer to the natural state of illness, but the time required to achieve modeling is generally longer [16]. Hydrocortisone administration induces a common pathological model for KYDS studies. In this model, the exogenous adrenal glucocorticoid intake is used to inhibit the gene expression of corticosteroid synthetase and inhibit ATCH secretion in the pituitary, thereby interfering with the function of the HPA axis and inducing pathological KYDS symptoms [17, 18]. However, an excessive intake of hydrocortisone leads to abnormal HPA axis functioning, inhibits the immune system, and affects other endocrine functions. At the same time, the modeling dose of hydrocortisone is hard to control. An excessive dosage may make it difficult to reflect KYDS symptoms; on the contrary, it will increase the hormone level in animals and produce the syndrome of kidney-yin deficiency, similar to adrenal cortex hyperfunction [19].

Our previous studies found that the fascicular zone of the adrenal cortex of Lewis rats was slightly thinned and mitochondria were reduced, slightly deformed, and swollen; in hypothalamic neurons, the somatic nuclei were large and irregular, mitochondria were significantly reduced, synapses of neurons were reduced, and secretory vesicles in the anterior part of synapses were reduced [13]. There are also other studies showing that Lewis rats showed lower corticosterone levels than Fischer animals, and Lewis rats had a central nervous system defect in biosynthesis of corticotropin releasing hormone [20, 21]. In this study, compared with Wistar rats, Lewis rats had obvious abnormal phenomena including a lower body temperature and inadequate grasp without any human intervention as well as poor mobility and memory in the open-field and MWM tests. This reflected the sensation of coldness, weakness, and mental depression of Lewis rats, consistent with the manifestation of KYDS [22]. Detecting HPA function has proven to be advantageous in that Lewis rats can spontaneously produce the pathological manifestations of KYDS. Accordingly, the adrenal index of Lewis rats was significantly lower; also, serum ACTH, CRH, cGMP, CORT, and urine 17-OHCS levels decreased significantly as did hypothalamic CRH expression. These results suggest spontaneous hypofunction of the HPA axis in Lewis rats without intervention.

The HPA axis plays an important role in maintaining the structural stability of the central nervous system. Abnormal glycolysis and gluconeogenesis can cause an energy metabolism disorder and lead to a state of “exhaustion,” similar to KYDS, caused by an HPA imbalance [18]. Differences in physiological characteristics between Lewis and Wistar rats show that Lewis rats offer a spontaneous animal model of KYDS because of their cold sensations, weakness, fatigue, and discernibly deficient HPA axis function. In contrast, HSF administration can increase cold sensations in Lewis rats, enhance their grasp, and make them more energetic. The HPA axis participates in immune system regulation by secreting neurotransmitters and various endocrine hormones; the immune system also acts on the HPA axis through the feedback of cytokines and hormone-like substances, 2 systems that communicate and regulate each other through bidirectional interactions to maintain the stability of the internal environment [23]. Hence, there is a complex bidirectional regulation between the immune system and the endocrine system [24]. Corticosteroids can inhibit the production of T helper (Th)1 cells when HPA axis function is low. Th1 cells secrete IL-2, INF-*γ*, and TNF-*α*; these can stimulate B lymphocytes to produce a large number of autoantibodies and induce the production of TGF-*β*, thus making the body susceptible to autoimmune diseases [25, 26]. It is clear that TNF-*α* and IFN-*γ* expression in the hypothalamus of Lewis rats decreased significantly, while IL-2 expression in the hypothalamus increased significantly. Administering HSF treatment to KYDS rats accelerated immune system activation related to the HPA axis function imbalance.

Modern mechanism research about KYDS involved the disfunction of immune, endocrine, nerve, and other multisystem functions, which showed as a complex and multidimensional molecular network imbalance. Studies concerning KYDS and immune system are particularly important. Studies showed that the number of T lymphocyte in the KYDS patient is significantly reduced and the B cells is abnormally activated, resulting in a series manifestation of KYDS such as weakness of the waist and knees, fatigue, and feeling cold throughout the body [27, 28]. Therefore, it indicated that one of the basic pathogenesis characteristics of KYDS was the imbalance of immune homeostasis, and it is important to improve the immune system balance in the treatment of KYDS patients.

## 5. Conclusions

Lewis rats spontaneously develop KYDS symptoms without any intervention. HSF administration can ameliorate KYDS behaviors and enhance the HPA axis function by increasing the levels of CRH, ACTH, and CORT in serum and 17-OHCS in urine. At the same time, HSF administration can significantly improve the expression of TNF-*α*, IFN-*γ*, and IL-2, secreted by Th1 cells, which might accelerate the immune system activation related to the HPA axis function. Consequently, HSF administration can alleviate the symptoms of KYDS; moreover, further studies need to explore the molecular mechanisms.

## Figures and Tables

**Table 1:** The primer sequences of the genes.

No.	Gene	Primer sequences (5′ ⟶ 3′)
1	TNF-*α*	F-5′-ATACACTGGCCCGAGGCAAC R-5′-CCACATCTCGGATCATGCTTTC
2	IFN-*γ*	F-5′-GGTGAACAACCCACAGATCC R-5′-CAGAATCAGCACCGACTCCT
3	IL-10	F-5′-AGTGGAGCAGGTGAAGAATGA R-5′-CACGTAGGCTTCTATGCAGTTG
4	CRH	F-5′-AATCTGCCACTCATTGCTCA R-5′-TAATCGCCCTTTCGGACAT
5	MR	F-5′-GGACAGAGTTGGCAGAGGTT R-5′-CTCGGAAGGTGTAGAAGCAGA
6	GR	F-5′-CCGTGGATGATGGTTACAGA R-5′-CGGGCTTGGTTGCTATCTC
7	GAPDH	F-5′-GGCACAGTCAAGGCTGAGAATG R-5′-ATGGTGGTGAAGACGCCAGTA
8	IL-2	F-5′-GGCTTTTGGCTTCATCATCT R-5′-CTTGTCCCTCTCCAGCACTT

## Data Availability

The datasets used and/or analyzed during the current study are available from the corresponding author upon reasonable request.

## Abbreviations

KYDS: Kidney-yang deficiency syndrome
HSF: *Hirsutella sinensis* fungus
BA: Bilateral total adrenalectomy
HYD: Hydrocortisone
HPA: Hypothalamic-pituitary-adrenal
MWM: Morris water maze
ELISA: Enzyme-linked immunoabsorbent assay
cDNA: Complementary deoxyribonucleic acid
qPCR: Quantitative polymerase chain reaction
ACTH: Adrenocorticotropic hormone
CORT: Corticosterone
CRH: Corticotropin releasing hormone
cAMP: Cyclic adenosine monophosphate
cGMP: Cyclic guanosine monophosphate
17-OHCS: 17-Hydroxy-cortico-steroid
GR: Glucocorticoid receptor
MR: Mineralocorticoid receptor
TNF: Tumor necrosis factor
IL: Interleukin.

## References

[1] Chen Q. (1993). *Experimental Methodology of Pharmacological Research in Traditional Chinese Medicine*.

[2] Shen Z. (1999). The location of deficiency syndrome of kidney yang. *Chinese Medical Journal*.

[3] Ji B., Li Y., Yang W. (2018). Jinkui shenqi pills ameliorate asthma with “kidney yang deficiency” by enhancing the function of the hypothalamic-pituitary-adrenal axis to regulate T helper 1/2 imbalance. *Evidence-based complementary and alternative medicine: ECAM*.

[4] Oitzl M. S., van Haarst A. D., Sutanto W., Ron de Kloet E. (1995). Corticosterone, brain mineralocorticoid receptors (MRs) and the activity of the hypothalamic-pituitary-adrenal (HPA) axis: the Lewis rat as an example of increased central MR capacity and a hyporesponsive HPA axis. *Psychoneuroendocrinology*.

[5] Yue K., Ye M., Zhou Z., Sun W., Lin X. (2013). The genusCordyceps: a chemical and pharmacological review. *Journal of Pharmacy and Pharmacology*.

[6] Chen Y.-C., Chen Y.-H., Pan B.-S., Chang M.-M., Huang B.-M. (2017). Functional study of Cordyceps sinensis and cordycepin in male reproduction: a review. *Journal of Food and Drug Analysis*.

[7] Lv M., Lv G. (2017). Research progress on chemical constituents and pharmacological activities of two types of mycelium from Cordyceps powder. *Chinese Traditional & Herbal Drugs*.

[8] Huang T., Lai H., Ko Y. (2015). Hirsutella sinensis mycelium attenuates bleomycin-induced pulmonary inflammation and fibrosis in vivo. *Scientific Reports*.

[9] Huang T., Chong K., Ojcius D. (2013). Hirsutella sinensis mycelium suppresses interleukin-1beta and interleukin-18 secretion by inhibiting both canonical and non-canonical inflammasomes. *Scientific Reports*.

[10] Ge F., Gui L., Li W. (2008). Study on effects of *Hirsutella sinensis* fermented mycelia on immunologic function in mice. *Chinese Journal of Clinical Pharmacology & Therapeutics*.

[11] Shou Q., Fu H., Zhang L. (2012). Study on treatment effect and mechanism of Hirsutella sinensis mycelium in idiopathic pulmonary fibrosis in rats. *China Journal of Chinese Materia Medica*.

[12] Soto-Tinoco E., Guerrero-Vargas N. N., Buijs R. M. (2016). Interaction between the hypothalamus and the immune system. *Experimental Physiology*.

[13] Shou Q., Zhang L., Cai Y. (2015). Kidney yang deficiency constitution and hypothalamicpituitary-adrenal axis dysfunction in Lewis rats. *Chinese Journal of Comparative Medicine*.

[14] Sneddon L. U., Halsey L. G., Bury N. R. (2017). Considering aspects of the 3Rs principles within experimental animal biology. *The Journal of Experimental Biology*.

[15] Proctor D. N., Balagopal P., Nair K. S. (1998). Age-related sarcopenia in humans is associated with reduced synthetic rates of specific muscle proteins. *The Journal of Nutrition*.

[16] Chen Y., Luo J., Xu Y. (2018). Evaluation and progress of modeling method about kidney yang deficiency animal model. *Chinese Archives of Traditional Chinese Medicine*.

[17] Pan Z., Liang L., Wang X. (2017). Study on the characteristics of mice model with deficiency syndrome induced by hydrocortisone and changes of adrenocortical function. *Shanghai Journal of Traditional Chinese Medicine*.

[18] Chen M., Zhao L., Jia W. (2005). Metabonomic study on the biochemical profiles of a hydrocortisone-induced animal model. *Journal of Proteome Research*.

[19] Yang Y., Li Z. (2007). Recent research on animal model of kidney-yang deficiency and its diagnostic indicators. *Journal of Liaoning University of Traditional Chinese Medicine*.

[20] Grota L. J., Bienen T., Felten D. L. (1997). Corticosterone responses of adult Lewis and Fischer rats. *Journal of Neuroimmunology*.

[21] Sternberg E. M., Young W. S., Bernardini R. (1989). A central nervous system defect in biosynthesis of corticotropin-releasing hormone is associated with susceptibility to streptococcal cell wall-induced arthritis in Lewis rats. *Proceedings of the National Academy of Sciences*.

[22] Ma C., Ye H., Lin Z. (2019). Evaluation of four kidney-yang deficiency models. *Jilin Journal of Traditional Chinese Medicine*.

[23] Dunn A. (2008). The HPA Axis and the immune system: a perspective. *Neuroimmune Biology*.

[24] Ottaviani E., Franchini A., Genedani S. (1999). ACTH and its role in immune-neuroendocrine functions. A comparative study. *Current Pharmaceutical Design*.

[25] Hofstetter H. H., Shive C. L., Forsthuber T. G. (2002). Pertussis toxin modulates the immune response to neuroantigens injected in incomplete Freund’s adjuvant: induction of Th1 cells and experimental autoimmune encephalomyelitis in the presence of high frequencies of Th2 cells. *The Journal of Immunology*.

[26] Bellavance M., Rivest S. (2014). The HPA-immune Axis and the immunomodulatory actions of glucocorticoids in the brain. *Frontiers in Immunology*.

[27] Cao B., Zhou Q., Wang C., Bao Y. (2015). Correlation between TCM patterns of chronic glomerulonephritis and immunological parameters. *Shanghai Journal of Traditional Chinese Medicine*.

[28] Xin G., Shi W., Xu L.-X., Su Y., Yan L.-J., Li K.-S. (2013). Serum BAFF is elevated in patients with IgA nephropathy and associated with clinical and histopathological features. *Journal of Nephrology*.

